# 3,3′-Dibenzyl-1,1′-[naphthalene-1,4-diylbis(methyl­ene)]di(1*H*-imidazol-3-ium) bis­(hexa­fluoro­phosphate)

**DOI:** 10.1107/S1600536811032132

**Published:** 2011-08-17

**Authors:** Chang-Lu Liu, Kun Huang

**Affiliations:** a Department of Chemistry and Chemical Engineering, Sichuan University of Arts and Science, Dazhou 635000, People’s Republic of China

## Abstract

In the title *N*-heterocyclic carbene compound, C_32_H_30_N_4_^2+^·2PF_6_^−^, the mean plane of the naphthalene ring system makes dihedral angles of 79.15 (15) and 76.85 (16) with the imidazole rings and 56.15 (19) and 80.56 (16)° with the benzene rings. An intra­molecular C—H⋯N hydrogen bond occurs. The crystal structure is stabilized by C—H⋯F inter­actions. In addition, π–π inter­actions [centroid–centroid distances = 3.848 (1) and 3.574 (3) Å] are observed. The nine equatorial F atoms in the two PF_6_^−^ anions were disordered over two positions with occupancy ratios of 0.545 (10):0.455 (10) and 0.793 (11):0.207 (11) in the two anions.

## Related literature

For the first free carbenes isolated, see: Arduengo *et al.* (1991 ▶). For the application of *N*-heterocyclic carbene ligands in transmetalation, see: Lin *et al.* (2009 ▶); Saito *et al.* (2011 ▶); Wang *et al.* (2005 ▶). For the synthesis of the title compound, see: Saito *et al.* (2011 ▶). For related structures, see: Saito *et al.* (2011 ▶). For standard bond lengths, see: Allen *et al.* (1987 ▶).
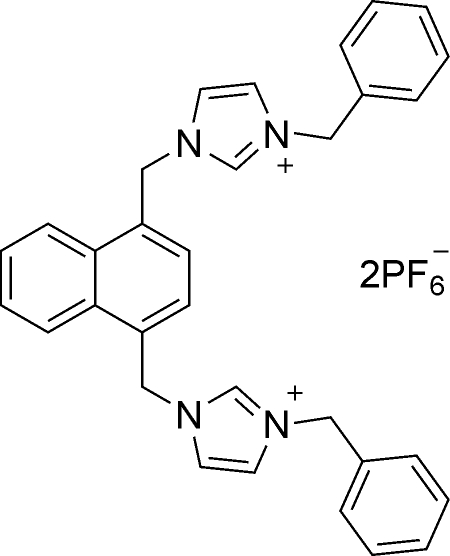


## Experimental

### 

#### Crystal data

C_32_H_30_N_4_^2+^·2PF_6_^−^
*M* *_r_* = 760.54
Monoclinic, 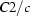

*a* = 33.8250 (9) Å
*b* = 11.6062 (3) Å
*c* = 17.6986 (5) Å
β = 101.158 (1)°
*V* = 6816.8 (3) Å^3^
*Z* = 8
Mo *K*α radiation
μ = 0.22 mm^−1^
*T* = 296 K
0.20 × 0.20 × 0.15 mm

#### Data collection

Bruker SMART CCD area-detector diffractometer
Absorption correction: multi-scan (*SADABS*; Bruker, 2001 ▶) *T*_min_ = 0.957, *T*_max_ = 0.967
5990 measured reflections
5990 independent reflections
4848 reflections with *I* > 2σ(*I*)
*R*_int_ = 0.029

#### Refinement

*R*[*F* ^2^ > 2σ(*F* ^2^)] = 0.067
*wR*(*F* ^2^) = 0.163
*S* = 1.04
5990 reflections
534 parameters
106 restraints
H-atom parameters constrained
Δρ_max_ = 0.43 e Å^−3^
Δρ_min_ = −0.25 e Å^−3^

### 

Data collection: *SMART* (Bruker, 2007 ▶); cell refinement: *SAINT* (Bruker, 2007 ▶); data reduction: *SAINT*; program(s) used to solve structure: *SHELXS97* (Sheldrick, 2008 ▶); program(s) used to refine structure: *SHELXL97* (Sheldrick, 2008 ▶); molecular graphics: *SHELXTL* (Sheldrick, 2008 ▶); software used to prepare material for publication: *SHELXTL*.

## Supplementary Material

Crystal structure: contains datablock(s) global, I. DOI: 10.1107/S1600536811032132/su2293sup1.cif

Structure factors: contains datablock(s) I. DOI: 10.1107/S1600536811032132/su2293Isup2.hkl

Supplementary material file. DOI: 10.1107/S1600536811032132/su2293Isup3.cml

Additional supplementary materials:  crystallographic information; 3D view; checkCIF report

## Figures and Tables

**Table 1:** Hydrogen-bond geometry (Å, °)

*D*—H⋯*A*	*D*—H	H⋯*A*	*D*⋯*A*	*D*—H⋯*A*
C1—H1⋯F3^i^	0.93	2.41	3.229 (6)	147
C7—H7*A*⋯F1^ii^	0.97	2.54	3.375 (5)	144
C7—H7*B*⋯F2^iii^	0.97	2.47	3.184 (5)	130
C9—H9⋯F4	0.93	2.45	3.358 (6)	164
C10—H10⋯F7	0.93	2.47	3.384 (10)	166
C14—H14⋯F10^iv^	0.93	2.55	3.453 (11)	165
C18—H18⋯F11	0.93	2.46	3.252 (12)	143
C18—H18⋯N3	0.93	2.62	3.102 (4)	113
C23—H23⋯F4	0.93	2.37	3.241 (6)	156
C24—H24⋯F9^i^	0.93	2.53	3.258 (15)	136
C25—H25⋯F2^v^	0.93	2.49	3.374 (6)	158

Symmetry codes: (i) 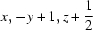; (ii) 
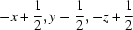
; (iii) 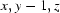; (iv) 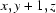; (v) 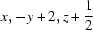.

**Fig. 1.**
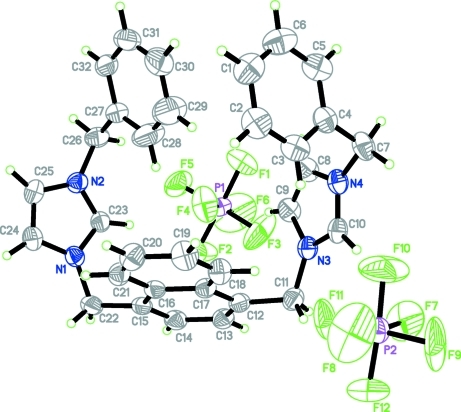
A view of the molecular structure of the title compound with atom numbering. The displacement ellipsoids are drawn at the 30% probability level. Only the major components of the disordered fluorine atoms are shown.

**Fig. 2.**
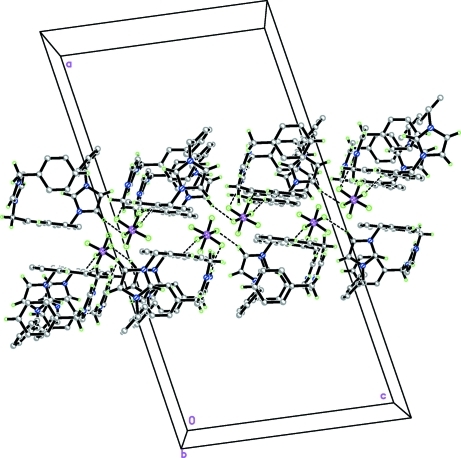
Crystal packing of the title compound viewed along the b axis, showing the C—H···F interactions (dashed lines) involving the cations and anions [H atoms not involved in these interactions have been omitted for clarity].

## supplementary crystallographic information

### Comment

Two decades have passed since the first free N-heterocyclic carbene (NHC) was disclosed by Arduengo and coworks (Arduengo *et al.*, 1991). 1,3-disubstituted imidazolium salts play important roles in synthesis of transition metal NHC's (Lin *et al.*, 2009; Saito *et al.*, 2011; Wang *et al.*, 2005). Herein, we report on the crystal structure of the title compound, a new NHC precursor.

The molecular structure of the title compound is shown in Fig. 1. Bond lengths (Allen *et al.*, 1987) and angles in the cation are normal. The mean plane of the naphthalene ring [A = (N1,N2,C23—C24)] makes dihedral angles with the mean planes of the imidazole rings [B = (N1,N2,C23—C25); C = (N3,N4,C8—C10)] and the benzene rings [D = (C27—C32); E = (C1—C6)] of A/B = 79.15 (15) °, A/C = 76.85 (16) °, A/D = 56.15 (19) °, and A/E = 80.56 (16)°. The PF_6_^-^ anions are disordered with two postitions found for nine F atoms (F4—F6, F7—F12) in the equitorial planes.

In the crystal there are weak π···π interactions involving the imidazole, benzene and naphthalene rings with centroid-centroid distances, *Cg*1···*Cg*3^i^, *Cg*5···*Cg*5^ii^ of 3.847 (2) and 3.5744 (19) Å, respectively [symmetry codes: (i) *x*, *y* + 1, *z*; (ii) -*x*, *y*, -*z* + 1/2. *Cg*1 centroid of the imidazole ring (N1,N2,C23—C24); *Cg*3 centroid of ring (C1—C6); *Cg*5 centroid of ring (C16—C21)]. In addition, a number of C—H···F hydrogen bonds are observed (Table 1 and Fig. 2).

### Experimental

The title compound was prepared according to the reported procedures (Saito *et al.*, 2011). Colourless single crystals suitable for X-ray diffraction were obtained by recrystallization from acetonitrile and ethyl ether (*v*/*v* = 1:1).

### Refinement

H atoms were placed in calculated orientations and treated as riding atoms: C—H = 0.93 and 0.97 Å, for CH and CH_2_ H-atoms, respectively, with *U*_iso_(H) =1.2*U*_eq_(C). Nine equatorial F atoms (F4—F6, F7—F12) in the two PF_6_^-^ anions were disordered over two positions (occupancies: 0.793 (11) and 0.207 (11) for F4—F6 and F4'-F6', respectively; 0.545 (10) and 0.455 (10) for F7—F12 and F7'-F12', respectively).

### Crystal data

**Table:**

C_32_H_30_N_4_^2+^·2PF_6_^−^	*F*(000) = 3104
*M**_r_* = 760.54	*D*_x_ = 1.482 Mg m^−^^3^
Monoclinic, *C*2/*c*	Mo *K*α radiation, λ = 0.71073 Å
Hall symbol: -C 2yc	Cell parameters from 9587 reflections
*a* = 33.8250 (9) Å	θ = 2.4–27.5°
*b* = 11.6062 (3) Å	µ = 0.22 mm^−^^1^
*c* = 17.6986 (5) Å	*T* = 296 K
β = 101.158 (1)°	Block, colourless
*V* = 6816.8 (3) Å^3^	0.20 × 0.20 × 0.15 mm
*Z* = 8	

### Data collection

**Table:**

Bruker SMART CCD area-detector diffractometer	5990 independent reflections
Radiation source: fine-focus sealed tube	4848 reflections with *I* > 2σ(*I*)
graphite	*R*_int_ = 0.029
φ and ω scans	θ_max_ = 25.0°, θ_min_ = 1.9°
Absorption correction: multi-scan (*SADABS*; Bruker, 2001)	*h* = −40→39
*T*_min_ = 0.957, *T*_max_ = 0.967	*k* = 0→13
5990 measured reflections	*l* = 0→20

### Refinement

**Table:**

Refinement on *F*^2^	Primary atom site location: structure-invariant direct methods
Least-squares matrix: full	Secondary atom site location: difference Fourier map
*R*[*F*^2^ > 2σ(*F*^2^)] = 0.067	Hydrogen site location: inferred from neighbouring sites
*wR*(*F*^2^) = 0.163	H-atom parameters constrained
*S* = 1.04	*w* = 1/[σ^2^(*F*_o_^2^) + (0.0576*P*)^2^ + 12.5479*P*] where *P* = (*F*_o_^2^ + 2*F*_c_^2^)/3
5990 reflections	(Δ/σ)_max_ = 0.004
534 parameters	Δρ_max_ = 0.43 e Å^−^^3^
106 restraints	Δρ_min_ = −0.25 e Å^−^^3^

### Special details

**Table:**

Geometry. Bond distances, angles *etc*. have been calculated using the rounded fractional coordinates. All su's are estimated from the variances of the (full) variance-covariance matrix. The cell e.s.d.'s are taken into account in the estimation of distances, angles and torsion angles
Refinement. Refinement of *F*^2^ against ALL reflections. The weighted *R*-factor *wR* and goodness of fit *S* are based on *F*^2^, conventional *R*-factors *R* are based on *F*, with *F* set to zero for negative *F*^2^. The threshold expression of *F*^2^ > σ(*F*^2^) is used only for calculating *R*-factors(gt) *etc*. and is not relevant to the choice of reflections for refinement. *R*-factors based on *F*^2^ are statistically about twice as large as those based on *F*, and *R*- factors based on ALL data will be even larger.

### Fractional atomic coordinates and isotropic or equivalent isotropic displacement parameters (Å^2^)

**Table:**

	*x*	*y*	*z*	*U*_iso_*/*U*_eq_	Occ. (<1)
N1	0.08264 (7)	0.9657 (2)	0.37930 (14)	0.0574 (9)	
N2	0.14129 (8)	0.9374 (2)	0.44984 (15)	0.0617 (9)	
N3	0.11786 (8)	0.5263 (2)	0.15155 (14)	0.0592 (9)	
N4	0.15515 (8)	0.3862 (2)	0.20370 (16)	0.0632 (10)	
C1	0.15298 (16)	0.2279 (4)	0.4641 (3)	0.0944 (19)	
C2	0.12019 (14)	0.2474 (4)	0.4077 (3)	0.0909 (17)	
C3	0.12467 (12)	0.2595 (3)	0.3324 (2)	0.0790 (14)	
C4	0.16251 (11)	0.2531 (3)	0.3133 (2)	0.0694 (11)	
C5	0.19513 (12)	0.2329 (3)	0.3711 (3)	0.0883 (17)	
C6	0.19023 (15)	0.2197 (4)	0.4466 (3)	0.1003 (17)	
C7	0.16755 (12)	0.2689 (3)	0.2311 (2)	0.0781 (16)	
C8	0.17466 (11)	0.4866 (3)	0.2285 (2)	0.0734 (14)	
C9	0.15153 (10)	0.5735 (3)	0.1960 (2)	0.0684 (11)	
C10	0.12119 (10)	0.4129 (3)	0.15704 (18)	0.0620 (11)	
C11	0.08189 (11)	0.5901 (3)	0.11210 (18)	0.0682 (11)	
C12	0.07110 (9)	0.6843 (3)	0.16312 (17)	0.0577 (10)	
C13	0.07231 (10)	0.7958 (3)	0.14081 (18)	0.0644 (11)	
C14	0.06459 (10)	0.8860 (3)	0.18869 (19)	0.0659 (12)	
C15	0.05548 (9)	0.8652 (3)	0.25894 (18)	0.0578 (10)	
C16	0.05272 (8)	0.7490 (3)	0.28405 (17)	0.0539 (10)	
C17	0.06026 (9)	0.6572 (3)	0.23542 (17)	0.0555 (10)	
C18	0.05654 (10)	0.5432 (3)	0.2607 (2)	0.0685 (12)	
C19	0.04682 (11)	0.5197 (3)	0.3299 (2)	0.0804 (16)	
C20	0.03969 (11)	0.6100 (4)	0.3780 (2)	0.0758 (14)	
C21	0.04222 (9)	0.7207 (3)	0.35529 (19)	0.0647 (11)	
C22	0.04995 (10)	0.9646 (3)	0.31026 (19)	0.0689 (11)	
C23	0.12086 (9)	0.9425 (3)	0.37841 (18)	0.0597 (11)	
C24	0.07865 (11)	0.9759 (3)	0.45441 (19)	0.0667 (12)	
C25	0.11500 (11)	0.9584 (3)	0.49870 (19)	0.0686 (11)	
C26	0.18335 (10)	0.8995 (4)	0.4714 (2)	0.0794 (14)	
C27	0.18627 (11)	0.7727 (4)	0.48956 (19)	0.0740 (14)	
C28	0.16502 (17)	0.6915 (4)	0.4413 (3)	0.126 (2)	
C29	0.1689 (2)	0.5760 (5)	0.4574 (3)	0.145 (3)	
C30	0.19425 (18)	0.5390 (5)	0.5218 (3)	0.118 (3)	
C31	0.21537 (13)	0.6164 (5)	0.5702 (3)	0.100 (2)	
C32	0.21155 (11)	0.7333 (4)	0.5550 (2)	0.0830 (16)	
P2	0.01796 (3)	0.22512 (7)	0.09338 (5)	0.0606 (3)	
F7	0.0471 (3)	0.2620 (10)	0.0432 (4)	0.150 (5)	0.545 (10)
F8	−0.0064 (5)	0.1822 (15)	0.1482 (8)	0.218 (7)	0.545 (10)
F9	0.0099 (3)	0.1149 (13)	0.0461 (12)	0.211 (7)	0.545 (10)
F10	0.0551 (4)	0.1765 (9)	0.1469 (9)	0.194 (6)	0.545 (10)
F11	0.0261 (2)	0.3391 (8)	0.1372 (8)	0.126 (4)	0.545 (10)
F12	−0.0194 (3)	0.2793 (10)	0.0455 (8)	0.176 (5)	0.545 (10)
F7'	0.0197 (5)	0.3549 (9)	0.0831 (11)	0.182 (7)	0.455 (10)
F8'	−0.0224 (3)	0.2198 (11)	0.1211 (10)	0.139 (6)	0.455 (10)
F9'	0.0171 (4)	0.0931 (7)	0.0937 (9)	0.145 (5)	0.455 (10)
F10'	0.0597 (3)	0.2222 (14)	0.0719 (12)	0.176 (7)	0.455 (10)
F11'	0.0399 (6)	0.246 (2)	0.1736 (5)	0.221 (9)	0.455 (10)
F12'	−0.0018 (5)	0.2177 (15)	0.0093 (5)	0.173 (6)	0.455 (10)
P1	0.19770 (3)	0.92074 (8)	0.20869 (5)	0.0678 (3)	
F1	0.23389 (10)	0.8405 (3)	0.2363 (2)	0.1598 (16)	
F2	0.16208 (12)	1.0041 (3)	0.1833 (3)	0.198 (2)	
F3	0.17942 (15)	0.8392 (3)	0.14383 (19)	0.190 (2)	
F4	0.17314 (15)	0.8443 (4)	0.2571 (3)	0.126 (2)	0.793 (11)
F5	0.2123 (2)	0.9987 (4)	0.2799 (3)	0.144 (3)	0.793 (11)
F6	0.2185 (3)	0.9949 (9)	0.1582 (5)	0.224 (5)	0.793 (11)
F4'	0.1816 (8)	0.937 (3)	0.2751 (11)	0.196 (13)	0.207 (11)
F5'	0.2303 (5)	1.015 (2)	0.230 (2)	0.178 (12)	0.207 (11)
F6'	0.2236 (9)	0.916 (2)	0.1454 (17)	0.178 (12)	0.207 (11)
H1	0.14990	0.22020	0.51500	0.1130*	
H2	0.09470	0.25260	0.41990	0.1090*	
H3	0.10210	0.27200	0.29400	0.0940*	
H5	0.22080	0.22810	0.35960	0.1060*	
H6	0.21250	0.20520	0.48530	0.1200*	
H7A	0.19550	0.25660	0.22780	0.0940*	
H7B	0.15130	0.21250	0.19850	0.0940*	
H8	0.19940	0.49320	0.26180	0.0880*	
H9	0.15730	0.65160	0.20250	0.0820*	
H10	0.10240	0.36020	0.13180	0.0740*	
H11A	0.05940	0.53740	0.09870	0.0820*	
H11B	0.08720	0.62350	0.06480	0.0820*	
H13	0.07840	0.81270	0.09300	0.0770*	
H14	0.06570	0.96170	0.17200	0.0790*	
H18	0.06090	0.48250	0.22900	0.0820*	
H19	0.04490	0.44370	0.34540	0.0970*	
H20	0.03320	0.59390	0.42560	0.0910*	
H21	0.03690	0.77970	0.38740	0.0780*	
H22A	0.05020	1.03630	0.28220	0.0830*	
H22B	0.02410	0.95790	0.32590	0.0830*	
H23	0.13160	0.93150	0.33440	0.0720*	
H24	0.05500	0.99210	0.47170	0.0800*	
H25	0.12130	0.96010	0.55220	0.0820*	
H26A	0.19670	0.94270	0.51600	0.0960*	
H26B	0.19720	0.91550	0.42940	0.0960*	
H28	0.14770	0.71560	0.39670	0.1510*	
H29	0.15400	0.52290	0.42410	0.1740*	
H30	0.19710	0.46060	0.53240	0.1410*	
H31	0.23270	0.59100	0.61440	0.1200*	
H32	0.22620	0.78570	0.58930	0.0990*	

### Atomic displacement parameters (Å^2^)

**Table:**

	*U*^11^	*U*^22^	*U*^33^	*U*^12^	*U*^13^	*U*^23^
N1	0.0562 (15)	0.0533 (15)	0.0632 (15)	0.0026 (12)	0.0131 (12)	−0.0050 (12)
N2	0.0576 (15)	0.0639 (17)	0.0618 (15)	−0.0065 (13)	0.0073 (12)	−0.0049 (13)
N3	0.0649 (16)	0.0524 (16)	0.0626 (15)	−0.0048 (12)	0.0183 (13)	−0.0055 (12)
N4	0.0701 (17)	0.0515 (16)	0.0719 (17)	−0.0020 (13)	0.0232 (14)	−0.0047 (13)
C1	0.117 (4)	0.082 (3)	0.084 (3)	−0.017 (3)	0.019 (3)	0.009 (2)
C2	0.097 (3)	0.084 (3)	0.099 (3)	−0.006 (2)	0.037 (3)	0.015 (2)
C3	0.073 (2)	0.075 (2)	0.090 (3)	−0.0007 (19)	0.018 (2)	0.013 (2)
C4	0.079 (2)	0.0464 (18)	0.085 (2)	−0.0022 (16)	0.0215 (19)	0.0030 (16)
C5	0.074 (3)	0.079 (3)	0.111 (3)	−0.007 (2)	0.016 (2)	0.010 (2)
C6	0.095 (3)	0.100 (3)	0.096 (3)	−0.018 (3)	−0.006 (3)	0.016 (3)
C7	0.096 (3)	0.051 (2)	0.094 (3)	0.0088 (18)	0.035 (2)	0.0017 (18)
C8	0.061 (2)	0.060 (2)	0.098 (3)	−0.0064 (17)	0.0125 (18)	−0.0120 (19)
C9	0.063 (2)	0.0527 (19)	0.090 (2)	−0.0108 (16)	0.0163 (18)	−0.0066 (17)
C10	0.073 (2)	0.0528 (19)	0.0645 (19)	−0.0110 (16)	0.0239 (17)	−0.0107 (15)
C11	0.080 (2)	0.067 (2)	0.0560 (18)	0.0009 (17)	0.0092 (16)	−0.0028 (16)
C12	0.0560 (18)	0.0601 (19)	0.0536 (17)	0.0000 (14)	0.0023 (13)	0.0000 (14)
C13	0.069 (2)	0.068 (2)	0.0545 (17)	0.0042 (16)	0.0075 (15)	0.0092 (15)
C14	0.070 (2)	0.055 (2)	0.068 (2)	0.0103 (16)	0.0018 (16)	0.0089 (16)
C15	0.0480 (16)	0.0588 (19)	0.0619 (18)	0.0072 (14)	−0.0010 (13)	−0.0019 (15)
C16	0.0405 (15)	0.0602 (18)	0.0576 (17)	0.0005 (13)	0.0014 (12)	−0.0029 (14)
C17	0.0492 (16)	0.0578 (19)	0.0571 (17)	−0.0045 (14)	0.0044 (13)	−0.0016 (14)
C18	0.073 (2)	0.058 (2)	0.077 (2)	−0.0109 (16)	0.0205 (17)	−0.0041 (17)
C19	0.087 (3)	0.067 (2)	0.093 (3)	−0.014 (2)	0.032 (2)	0.010 (2)
C20	0.077 (2)	0.085 (3)	0.070 (2)	−0.013 (2)	0.0255 (18)	0.002 (2)
C21	0.0586 (19)	0.073 (2)	0.0627 (19)	−0.0069 (16)	0.0122 (15)	−0.0082 (16)
C22	0.0598 (19)	0.065 (2)	0.077 (2)	0.0142 (16)	0.0009 (16)	−0.0039 (17)
C23	0.0579 (18)	0.062 (2)	0.0599 (18)	0.0012 (15)	0.0132 (15)	−0.0041 (15)
C24	0.069 (2)	0.063 (2)	0.073 (2)	−0.0040 (17)	0.0257 (18)	−0.0052 (17)
C25	0.083 (2)	0.069 (2)	0.0571 (18)	−0.0074 (18)	0.0215 (17)	−0.0061 (16)
C26	0.057 (2)	0.095 (3)	0.079 (2)	−0.0070 (19)	−0.0048 (17)	−0.007 (2)
C27	0.066 (2)	0.089 (3)	0.062 (2)	0.0104 (19)	−0.0002 (16)	−0.0031 (18)
C28	0.158 (5)	0.086 (3)	0.104 (3)	0.012 (3)	−0.050 (3)	−0.008 (3)
C29	0.189 (6)	0.084 (4)	0.132 (5)	0.017 (4)	−0.040 (4)	−0.013 (3)
C30	0.142 (5)	0.099 (4)	0.112 (4)	0.037 (3)	0.023 (3)	0.011 (3)
C31	0.082 (3)	0.143 (5)	0.076 (3)	0.041 (3)	0.019 (2)	0.027 (3)
C32	0.062 (2)	0.126 (4)	0.061 (2)	0.012 (2)	0.0122 (17)	−0.002 (2)
P2	0.0639 (5)	0.0547 (5)	0.0654 (5)	−0.0080 (4)	0.0180 (4)	−0.0066 (4)
F7	0.154 (9)	0.226 (11)	0.091 (4)	−0.023 (7)	0.079 (5)	0.025 (5)
F8	0.262 (15)	0.250 (14)	0.184 (9)	−0.128 (11)	0.148 (11)	0.007 (9)
F9	0.140 (8)	0.190 (11)	0.327 (15)	−0.072 (8)	0.104 (10)	−0.211 (11)
F10	0.179 (9)	0.119 (7)	0.234 (13)	0.039 (6)	−0.083 (9)	0.055 (7)
F11	0.098 (5)	0.096 (6)	0.199 (10)	−0.032 (4)	0.067 (6)	−0.091 (7)
F12	0.108 (5)	0.225 (10)	0.163 (9)	0.055 (6)	−0.052 (6)	−0.001 (8)
F7'	0.224 (13)	0.058 (5)	0.272 (16)	−0.011 (5)	0.066 (13)	0.018 (8)
F8'	0.068 (4)	0.125 (7)	0.241 (17)	−0.014 (4)	0.074 (6)	−0.067 (9)
F9'	0.141 (7)	0.052 (4)	0.234 (14)	0.006 (4)	0.016 (8)	0.056 (7)
F10'	0.067 (5)	0.176 (10)	0.302 (17)	−0.047 (6)	0.077 (8)	−0.168 (11)
F11'	0.270 (18)	0.32 (2)	0.057 (4)	−0.155 (15)	−0.011 (6)	−0.022 (8)
F12'	0.241 (14)	0.191 (12)	0.062 (4)	−0.058 (10)	−0.034 (5)	0.015 (5)
P1	0.0671 (6)	0.0703 (6)	0.0606 (5)	−0.0036 (4)	−0.0012 (4)	0.0045 (4)
F1	0.118 (2)	0.166 (3)	0.189 (3)	0.069 (2)	0.014 (2)	0.005 (3)
F2	0.156 (3)	0.117 (3)	0.268 (5)	0.044 (2)	−0.091 (3)	0.005 (3)
F3	0.270 (5)	0.171 (4)	0.106 (2)	−0.068 (3)	−0.018 (3)	−0.047 (2)
F4	0.164 (4)	0.092 (3)	0.146 (4)	−0.013 (3)	0.092 (3)	0.009 (2)
F5	0.181 (6)	0.097 (3)	0.124 (4)	0.006 (3)	−0.046 (4)	−0.043 (3)
F6	0.271 (10)	0.221 (9)	0.187 (7)	−0.097 (8)	0.065 (6)	0.100 (7)
F4'	0.21 (2)	0.30 (3)	0.119 (12)	−0.03 (2)	0.136 (14)	−0.061 (18)
F5'	0.055 (8)	0.21 (2)	0.26 (3)	−0.054 (10)	0.007 (13)	0.01 (2)
F6'	0.21 (2)	0.14 (2)	0.23 (2)	0.038 (19)	0.160 (19)	−0.010 (19)

### Geometric parameters (Å, °)

**Table:**

P2—F8'	1.539 (12)	C15—C22	1.502 (5)
P2—F9'	1.533 (8)	C15—C16	1.429 (5)
P2—F10'	1.532 (12)	C16—C17	1.423 (5)
P2—F11'	1.489 (11)	C16—C21	1.413 (4)
P2—F12'	1.511 (9)	C17—C18	1.410 (5)
P2—F11	1.531 (11)	C18—C19	1.356 (5)
P2—F12	1.515 (12)	C19—C20	1.400 (5)
P2—F7'	1.520 (11)	C20—C21	1.354 (6)
P2—F7	1.511 (9)	C24—C25	1.340 (5)
P2—F8	1.476 (16)	C26—C27	1.506 (6)
P2—F9	1.524 (17)	C27—C28	1.377 (6)
P2—F10	1.528 (14)	C27—C32	1.378 (5)
P1—F1	1.541 (4)	C28—C29	1.372 (7)
P1—F2	1.543 (4)	C29—C30	1.357 (8)
P1—F3	1.524 (4)	C30—C31	1.347 (8)
P1—F6	1.509 (10)	C31—C32	1.384 (7)
P1—F4	1.577 (5)	C1—H1	0.9300
P1—F5	1.552 (5)	C2—H2	0.9300
P1—F6'	1.55 (3)	C3—H3	0.9300
P1—F4'	1.40 (2)	C5—H5	0.9300
P1—F5'	1.55 (2)	C6—H6	0.9300
N1—C22	1.481 (4)	C7—H7B	0.9700
N1—C23	1.324 (4)	C7—H7A	0.9700
N1—C24	1.367 (4)	C8—H8	0.9300
N2—C23	1.320 (4)	C9—H9	0.9300
N2—C25	1.377 (4)	C10—H10	0.9300
N2—C26	1.467 (4)	C11—H11B	0.9700
N3—C10	1.323 (4)	C11—H11A	0.9700
N3—C9	1.367 (4)	C13—H13	0.9300
N3—C11	1.479 (4)	C14—H14	0.9300
N4—C10	1.315 (4)	C18—H18	0.9300
N4—C7	1.478 (4)	C19—H19	0.9300
N4—C8	1.369 (4)	C20—H20	0.9300
C1—C6	1.358 (8)	C21—H21	0.9300
C1—C2	1.360 (7)	C22—H22A	0.9700
C2—C3	1.377 (6)	C22—H22B	0.9700
C3—C4	1.388 (6)	C23—H23	0.9300
C4—C5	1.372 (6)	C24—H24	0.9300
C4—C7	1.508 (5)	C25—H25	0.9300
C5—C6	1.386 (7)	C26—H26A	0.9700
C8—C9	1.336 (5)	C26—H26B	0.9700
C11—C12	1.507 (5)	C28—H28	0.9300
C12—C13	1.356 (5)	C29—H29	0.9300
C12—C17	1.433 (4)	C30—H30	0.9300
C13—C14	1.403 (5)	C31—H31	0.9300
C14—C15	1.359 (5)	C32—H32	0.9300
			
F8—P2—F11	91.6 (8)	C14—C15—C22	119.6 (3)
F8—P2—F12	89.8 (8)	C15—C16—C21	122.7 (3)
F9—P2—F10	93.9 (7)	C17—C16—C21	118.1 (3)
F9—P2—F11	177.2 (9)	C15—C16—C17	119.2 (3)
F9—P2—F12	90.0 (7)	C12—C17—C18	122.9 (3)
F10—P2—F11	87.7 (6)	C16—C17—C18	118.2 (3)
F10—P2—F12	175.5 (7)	C12—C17—C16	118.9 (3)
F11—P2—F12	88.5 (6)	C17—C18—C19	121.8 (3)
F7'—P2—F8'	97.8 (8)	C18—C19—C20	119.9 (3)
F7'—P2—F9'	173.0 (9)	C19—C20—C21	120.1 (3)
F7'—P2—F10'	86.2 (9)	C16—C21—C20	121.8 (3)
F7'—P2—F11'	85.9 (11)	N1—C22—C15	110.2 (3)
F7'—P2—F12'	87.6 (10)	N1—C23—N2	109.2 (3)
F8'—P2—F9'	86.6 (7)	N1—C24—C25	107.7 (3)
F8'—P2—F10'	174.5 (9)	N2—C25—C24	106.9 (3)
F8'—P2—F11'	90.7 (10)	N2—C26—C27	111.6 (3)
F8'—P2—F12'	93.5 (9)	C26—C27—C28	122.1 (3)
F9'—P2—F10'	89.9 (8)	C26—C27—C32	120.6 (4)
F9'—P2—F11'	99.5 (11)	C28—C27—C32	117.3 (4)
F9'—P2—F12'	86.7 (9)	C27—C28—C29	121.5 (5)
F10'—P2—F11'	85.8 (11)	C28—C29—C30	120.2 (5)
F10'—P2—F12'	90.4 (10)	C29—C30—C31	119.6 (5)
F11'—P2—F12'	172.8 (11)	C30—C31—C32	120.8 (5)
F7—P2—F12	97.0 (6)	C27—C32—C31	120.5 (4)
F8—P2—F9	90.8 (9)	C2—C1—H1	120.00
F8—P2—F10	88.0 (8)	C6—C1—H1	120.00
F7—P2—F8	173.2 (7)	C3—C2—H2	120.00
F7—P2—F9	89.0 (7)	C1—C2—H2	120.00
F7—P2—F10	85.3 (6)	C2—C3—H3	120.00
F7—P2—F11	88.8 (6)	C4—C3—H3	120.00
F1—P1—F2	178.0 (2)	C4—C5—H5	120.00
F1—P1—F3	92.6 (2)	C6—C5—H5	120.00
F1—P1—F4	87.6 (2)	C5—C6—H6	120.00
F2—P1—F5	88.8 (3)	C1—C6—H6	120.00
F2—P1—F6	84.1 (4)	N4—C7—H7B	110.00
F2—P1—F4'	75.6 (12)	C4—C7—H7A	110.00
F2—P1—F5'	96.2 (8)	H7A—C7—H7B	108.00
F2—P1—F6'	109.3 (10)	C4—C7—H7B	110.00
F3—P1—F4	83.1 (2)	N4—C7—H7A	110.00
F3—P1—F5	173.1 (3)	N4—C8—H8	126.00
F3—P1—F6	94.4 (4)	C9—C8—H8	126.00
F3—P1—F4'	123.3 (12)	C8—C9—H9	126.00
F3—P1—F5'	142.1 (12)	N3—C9—H9	126.00
F3—P1—F6'	68.7 (10)	N3—C10—H10	125.00
F4—P1—F5	90.3 (3)	N4—C10—H10	125.00
F4—P1—F6	176.0 (4)	N3—C11—H11A	110.00
F5—P1—F6	92.1 (4)	N3—C11—H11B	110.00
F4'—P1—F5'	94.3 (17)	H11A—C11—H11B	108.00
F4'—P1—F6'	167.6 (16)	C12—C11—H11B	110.00
F5'—P1—F6'	74.1 (15)	C12—C11—H11A	110.00
F1—P1—F5	89.2 (3)	C12—C13—H13	119.00
F1—P1—F6	95.7 (4)	C14—C13—H13	119.00
F1—P1—F4'	103.3 (12)	C13—C14—H14	119.00
F1—P1—F5'	82.2 (8)	C15—C14—H14	119.00
F1—P1—F6'	71.5 (10)	C17—C18—H18	119.00
F2—P1—F3	89.4 (2)	C19—C18—H18	119.00
F2—P1—F4	92.7 (2)	C20—C19—H19	120.00
C22—N1—C24	127.2 (3)	C18—C19—H19	120.00
C23—N1—C24	108.0 (3)	C19—C20—H20	120.00
C22—N1—C23	124.3 (3)	C21—C20—H20	120.00
C23—N2—C25	108.2 (3)	C20—C21—H21	119.00
C23—N2—C26	124.4 (3)	C16—C21—H21	119.00
C25—N2—C26	127.0 (3)	N1—C22—H22A	110.00
C9—N3—C10	107.9 (3)	C15—C22—H22B	110.00
C9—N3—C11	126.1 (3)	H22A—C22—H22B	108.00
C10—N3—C11	125.7 (3)	N1—C22—H22B	110.00
C7—N4—C8	126.2 (3)	C15—C22—H22A	110.00
C7—N4—C10	125.5 (3)	N1—C23—H23	125.00
C8—N4—C10	108.0 (3)	N2—C23—H23	125.00
C2—C1—C6	120.4 (5)	C25—C24—H24	126.00
C1—C2—C3	120.0 (5)	N1—C24—H24	126.00
C2—C3—C4	120.6 (4)	C24—C25—H25	127.00
C5—C4—C7	121.0 (4)	N2—C25—H25	127.00
C3—C4—C7	120.7 (3)	N2—C26—H26A	109.00
C3—C4—C5	118.3 (3)	N2—C26—H26B	109.00
C4—C5—C6	120.6 (4)	C27—C26—H26B	109.00
C1—C6—C5	120.1 (5)	H26A—C26—H26B	108.00
N4—C7—C4	110.5 (3)	C27—C26—H26A	109.00
N4—C8—C9	107.4 (3)	C29—C28—H28	119.00
N3—C9—C8	107.4 (3)	C27—C28—H28	119.00
N3—C10—N4	109.4 (3)	C28—C29—H29	120.00
N3—C11—C12	110.7 (3)	C30—C29—H29	120.00
C11—C12—C13	119.6 (3)	C29—C30—H30	120.00
C11—C12—C17	120.6 (3)	C31—C30—H30	120.00
C13—C12—C17	119.8 (3)	C32—C31—H31	120.00
C12—C13—C14	121.1 (3)	C30—C31—H31	120.00
C13—C14—C15	121.5 (3)	C27—C32—H32	120.00
C14—C15—C16	119.5 (3)	C31—C32—H32	120.00
C16—C15—C22	120.9 (3)		
			
C23—N1—C22—C15	40.0 (4)	C11—C12—C13—C14	−176.5 (3)
C24—N1—C22—C15	−131.3 (3)	C17—C12—C13—C14	2.4 (5)
C22—N1—C23—N2	−172.6 (3)	C11—C12—C17—C16	176.1 (3)
C24—N1—C23—N2	0.1 (4)	C11—C12—C17—C18	−4.1 (5)
C22—N1—C24—C25	172.4 (3)	C13—C12—C17—C16	−2.8 (5)
C23—N1—C24—C25	−0.1 (4)	C13—C12—C17—C18	176.9 (3)
C25—N2—C23—N1	−0.1 (4)	C12—C13—C14—C15	−0.3 (5)
C26—N2—C23—N1	172.1 (3)	C13—C14—C15—C16	−1.5 (5)
C23—N2—C25—C24	0.0 (4)	C13—C14—C15—C22	176.3 (3)
C26—N2—C25—C24	−171.9 (3)	C14—C15—C16—C17	1.1 (4)
C23—N2—C26—C27	−93.1 (4)	C14—C15—C16—C21	−178.1 (3)
C25—N2—C26—C27	77.6 (4)	C22—C15—C16—C17	−176.7 (3)
C10—N3—C9—C8	−0.6 (4)	C22—C15—C16—C21	4.2 (5)
C11—N3—C9—C8	172.5 (3)	C14—C15—C22—N1	−112.9 (3)
C9—N3—C10—N4	0.8 (4)	C16—C15—C22—N1	64.8 (4)
C11—N3—C10—N4	−172.3 (3)	C15—C16—C17—C12	1.1 (4)
C9—N3—C11—C12	−40.9 (4)	C15—C16—C17—C18	−178.7 (3)
C10—N3—C11—C12	131.0 (3)	C21—C16—C17—C12	−179.8 (3)
C8—N4—C7—C4	68.7 (4)	C21—C16—C17—C18	0.5 (4)
C10—N4—C7—C4	−103.9 (4)	C15—C16—C21—C20	179.9 (3)
C7—N4—C8—C9	−173.3 (3)	C17—C16—C21—C20	0.7 (5)
C10—N4—C8—C9	0.3 (4)	C12—C17—C18—C19	179.0 (3)
C7—N4—C10—N3	172.9 (3)	C16—C17—C18—C19	−1.2 (5)
C8—N4—C10—N3	−0.7 (4)	C17—C18—C19—C20	0.8 (5)
C6—C1—C2—C3	−0.3 (7)	C18—C19—C20—C21	0.5 (6)
C2—C1—C6—C5	1.0 (7)	C19—C20—C21—C16	−1.2 (5)
C1—C2—C3—C4	−0.6 (6)	N1—C24—C25—N2	0.0 (4)
C2—C3—C4—C5	0.9 (5)	N2—C26—C27—C28	49.1 (5)
C2—C3—C4—C7	−178.7 (4)	N2—C26—C27—C32	−132.8 (3)
C3—C4—C5—C6	−0.2 (5)	C26—C27—C28—C29	178.0 (5)
C7—C4—C5—C6	179.3 (4)	C32—C27—C28—C29	−0.2 (7)
C3—C4—C7—N4	63.1 (4)	C26—C27—C32—C31	−177.6 (4)
C5—C4—C7—N4	−116.5 (4)	C28—C27—C32—C31	0.7 (6)
C4—C5—C6—C1	−0.7 (6)	C27—C28—C29—C30	−0.4 (9)
N4—C8—C9—N3	0.2 (4)	C28—C29—C30—C31	0.5 (9)
N3—C11—C12—C13	117.3 (3)	C29—C30—C31—C32	−0.1 (8)
N3—C11—C12—C17	−61.7 (4)	C30—C31—C32—C27	−0.6 (7)

### Hydrogen-bond geometry (Å, °)

**Table:**

*D*—H···*A*	*D*—H	H···*A*	*D*···*A*	*D*—H···*A*
C1—H1···F3^i^	0.93	2.41	3.229 (6)	147
C7—H7A···F1^ii^	0.97	2.54	3.375 (5)	144
C7—H7B···F2^iii^	0.97	2.47	3.184 (5)	130
C9—H9···F4	0.93	2.45	3.358 (6)	164
C10—H10···F7	0.93	2.47	3.384 (10)	166
C14—H14···F10^iv^	0.93	2.55	3.453 (11)	165
C18—H18···F11	0.93	2.46	3.252 (12)	143
C18—H18···N3	0.93	2.62	3.102 (4)	113
C23—H23···F4	0.93	2.37	3.241 (6)	156
C24—H24···F9^i^	0.93	2.53	3.258 (15)	136
C25—H25···F2^v^	0.93	2.49	3.374 (6)	158

Symmetry codes: (i) *x*, −*y*+1, *z*+1/2; (ii) −*x*+1/2, *y*−1/2, −*z*+1/2; (iii) *x*, *y*−1, *z*; (iv) *x*, *y*+1, *z*; (v) *x*, −*y*+2, *z*+1/2.

## References

[bb1] Allen, F. H., Kennard, O., Watson, D. G., Brammer, L., Orpen, A. G. & Taylor, R. (1987). *J. Chem. Soc., Perkin Trans. 2*, pp. S1–19.

[bb2] Arduengo, A. J., Harlow, R. L. & Kline, M. (1991). *J. Am. Chem. Soc.* **113**, 361—363.

[bb3] Bruker (2001). *SADABS* Bruker AXS Inc., Madison, Wisconsin, USA.

[bb4] Bruker (2007). *SMART* and *SAINT* Bruker AXS Inc., Madison, Wisconsin, USA.

[bb5] Lin, J. C. Y., Huang, R. T. W., Lee, C. S., Bhattacharyya, A., Hwang, W. S. & Lin, I. J. B. (2009). *Chem. Rev.* **109**, 3561—3598. 10.1021/cr8005153 19361198

[bb6] Saito, S., Saika, M., Yamasaki, R., Azumaya, I. & Masu, H. (2011). *Organometallics*, **30**, 1366–1373.

[bb7] Sheldrick, G. M. (2008). *Acta Cryst.* A**64**, 112–122. 10.1107/S0108767307043930 18156677

[bb8] Wang, J. W., Xu, F. B., Li, Q. S., Song, H. B. & Zhang, Z. Z. (2005). *Inorg. Chem. Commun.* **8**, 1053–1055.

